# Bacterial endophyte communities in the foliage of coast redwood and giant sequoia

**DOI:** 10.3389/fmicb.2015.01008

**Published:** 2015-09-22

**Authors:** Alyssa A. Carrell, Anna C. Frank

**Affiliations:** ^1^Life and Environmental Sciences, School of Natural Sciences, University of California, MercedMerced, CA, USA; ^2^Department of Biology, Duke UniversityDurham, NC, USA; ^3^Environmental Sciences Division, Oak Ridge National LaboratoryOak Ridge, TN, USA; ^4^Sierra Nevada Research Institute, University of California, MercedMerced, CA, USA

**Keywords:** bacterial endophytes, 16S rRNA, foliage, microbiome, giant sequoia, redwood, *Sequoia sempervirens*, *Sequoiadendron giganteum*

## Abstract

The endophytic bacterial microbiome, with an emerging role in plant nutrient acquisition and stress tolerance, is much less studied in natural plant populations than in agricultural crops. In a previous study, we found consistent associations between trees in the pine family and acetic acid bacteria (AAB) occurring at high relative abundance inside their needles. Our objective here was to determine if that pattern may be general to conifers, or alternatively, is more likely restricted to pines or conifers growing in nutrient limited and exposed environments. We used 16S rRNA pyrosequencing to characterize the foliar endophyte communities of two conifers in the *Cupressaceae* family: Two coast redwood (CR; *Sequoia sempervirens*) populations and one giant sequoia (GS; *Sequoiadendron giganteum*) population were sampled. Similar to the pines, the endophyte communities of the giant trees were dominated by Proteobacteria, Firmicutes, Acidobacteria, and Actinobacteria. However, although some major operational taxonomic units (OTUs) occurred at a high relative abundance of 10–40% in multiple samples, no specific group of bacteria dominated the endophyte community to the extent previously observed in high-elevation pines. Several of the dominating bacterial groups in the CR and GS foliage (e.g., *Bacillus*, *Burkholderia*, Actinomycetes) are known for disease- and pest suppression, raising the possibility that the endophytic microbiome protects the giant trees against biotic stress. Many of the most common and abundant OTUs in our dataset were most similar to 16S rRNA sequences from bacteria found in lichens or arctic plants. For example, an OTU belonging to the uncultured Rhizobiales LAR1 lineage, which is commonly associated with lichens, was observed at high relative abundance in many of the CR samples. The taxa shared between the giant trees, arctic plants, and lichens may be part of a broadly defined endophyte microbiome common to temperate, boreal, and tundra ecosystems.

## Introduction

The plant microbiome is essential to plant health (Turner et al., 2013; Berg, 2014; Peñuelas and Terradas, 2014), but the role of microbes colonizing most wild plants still remains unknown. For example, while a number of studies have examined the fungal endophyte communities inside the leaves of forest trees (Ganley et al., 2004; Arnold et al., 2007; Oono et al., 2014; Qadri et al., 2014), less is known about the role and diversity of their bacterial counterparts. The motivation for studying endophytic microbiomes comes mainly from studies of agricultural crops: Over the last two decades or so, a number of studies—most of them focused on bacterial isolates—have demonstrated that endophytes can benefit plants and crop yield through enhanced nutrient uptake, disease suppression, increased abiotic stress tolerance, and direct stimulation of plant growth, all from within the plant tissues (Rosenblueth and Martinez-Romero, 2006; Hardoim et al., 2008; Reinhold-Hurek and Hurek, 2011). In addition, a few studies on natural plant populations suggest that the bacterial endophytes associated with wild plants can affect plant traits, for example by fixing nitrogen (N), altering soil geochemical cycles to enable plant persistence, and producing compounds that are antagonistic against fungal pests (Adams et al., 2008; Anand et al., 2013; Rout et al., 2013; Knoth et al., 2014).

A better appreciation of how wild plants interact with their native microbiomes may be critical for understanding and predicting how terrestrial ecosystems will respond to current and projected global change (Rodriguez et al., 2004; Porras-Alfaro and Bayman, 2011; Berg, 2014). The coniferous forests in the Northern Hemisphere are potential major carbon (C) sinks, and their response to warming, elevated CO_2_, and increased disease pressure will influence the amount of C they can store. Many of the traits that influence this response can be microbially mediated, including defense, N-fixation, and abiotic stress tolerance (Friesen et al., 2011).

Community 16S rRNA sequencing can yield some insight into the relationship between a plant host and its associated microbiome, as well as detect endophyte community members with potential functional importance. Recent work on model-, agricultural-, and biofuel plants (e.g., *Arabidopsis, Oryza, Zea*, and *Populus*) suggests that bacterial endophyte communities are generally influenced by a combination of host species identity, host genotype, season, and environment, with substantial variation in taxonomic composition across plant individuals or species (Gottel et al., 2011; Bulgarelli et al., 2012; Lundberg et al., 2012; Shakya et al., 2013; Schlaeppi et al., 2014; Aleklett et al., 2015; Edwards et al., 2015; Müller et al., 2015; Shen and Fulthorpe, 2015). There are exceptions to this pattern, for example in *Sphagnum* mosses, where *Burkholderia* sp. dominate across individual plants as well as plant species, likely due to their vertical transmission (Bragina et al., 2013). Similarly, our recent study of limber pine (*Pinus flexilis*) and Engelmann spruce (*Picea engelmannii*) growing at high elevation (3000–3400 m), showed that their foliar endophyte microbiomes were consistently dominated by a few operational taxonomic units (OTUs) in the *Acetobacteraceae*, or acetic acid bacteria (AAB), a family of Alphaproteobacteria commonly associated with N_2_-fixation (Fuentes-Ramirez et al., 2001; Kersters et al., 2006; Dutta and Gachhui, 2007).

In order to determine whether the pattern we observed in the high elevation conifers—recurring dominance by a few endophytic taxa—is unique to trees in the pine family, and/or the extreme subalpine environment, or alternatively, is common to conifer species across habitats, we here explore the foliar bacterial endophytic communities of coast redwood (CR) and giant sequoia (GS).

Coast redwood and giant sequoia are the tallest and largest living tree species on Earth, respectively. The oldest known GS individuals are about 3,500 years old, and CR individuals have life spans that can extend 2000 years. Both are the only extant species in their respective genera, with extremely restricted distributions; CR occurs exclusively in the cloud-inundated humid areas along the coast of central and northern California; GS occurs in scattered groves along a narrow belt along the western Sierra Nevada, California, at elevations that generally range from 1400 to 2000 m. While fungal endophytes of CR have received some attention, to our knowledge, no studies of bacterial endophytes in CR or GS exist. The investigation of fungal endophytes in CR was pioneered by Carroll and Carroll (1978), who isolated four different endophyte species. A follow-up study that also examined spatial patterns in fungal endophyte communities found a higher diversity of fungal species (Espinosa-Garcia and Langenheim, 1990). The most extensive study to date documented 16 different endophyte species, and found that the fungal endophyte community was stable among host individuals and along a north to south distribution of CR, with dominance of *Pleuroplacoema* sp. (Rollinger and Langenheim, 1993).

Here, we used 16S rRNA pyrosequencing to characterize the taxonomic composition of bacteria in surface-sterilized foliage of two populations of CR (one in Northern California and one in Central California), and one population of GS. At each site, we sampled three individuals. To contrast inter- and intra tree variation in the endophytic community, we took samples from the lower, middle, and upper canopy of each tree.

## Materials and Methods

### Sample Collection and Sterilization

We collected CR needles from a Northern California site (Samuel P. Taylor State Park, Lagunitas) in November 2011 and a Central California site (Big Creek UC Natural Reserve, in Big Sur) in October 2011. We collected GS needles from trees growing at Freeman Creek Grove in Sequoia National Monument, Porterville, CA, USA in August 2011. To assess the difference in endophytic communities across individuals, locations, and species, we collected needles from three individuals trees in each of the three locations; GS trees A, B, and C from Freeman Creek Grove, CR trees D, E, and F from Big Creek, and trees G, I, and H from Samuel P. Taylor SP). To investigate intra-tree variation in the endophytic community, we sampled needles from three canopy heights (lower, middle, and upper) from each tree. For all downstream processing and analysis, we treated the resulting 27 samples individually (i.e., we did not pool them). For each sample, we removed approximately 10 g of needles with a sterile razor blade, placed them in a ziplock bag, and transported them to the University of California, Merced at 4°C. We sterilized the needles via submersion in ethanol for 1 min, 30% hydrogen peroxide for three minutes, followed by three rinses with sterile de-ionized water, and stored them at -20°C. We confirmed surface sterility of foliage by negative PCR amplification (but not sequencing) of the final rinse.

### DNA Extraction

We pulverized the needles to a fine powder using liquid nitrogen in a sterile mortar. We extracted DNA from 0.6 g of the pulverized tissue in a 2 ml screw cap tube containing 800 μl of CTAB solution (1 ml CTAB buffer, 0.04 g of polyvinylpyrrolidone, 5 μl of 2-mercaptoethanol), incubated in a dry bath at 60°C for 2 h, and then homogenized with 0.3 g of 0.11 mm sterile glass beads with a bead beater for 3 min. We removed proteins by adding an equal amount of chloroform and centrifuged the sample for 10 min at 16 rcf. We placed the aqueous top phase in a sterile 2 ml snap cap tube with 1/10 volume of cold 3 M sodium acetate and 1/2 volume cold isopropanol and placed it in a –20°C freezer overnight to precipitate the nucleic acids. We then centrifuged the sample for 30 min at 16 rcf, decanted the supernatant, added 700 μl of 70% ethanol, and centrifuged the sample for 10 min. We resuspended the air-dried pellet with 30 μl of DNA resuspension fluid (1.0 M Tris-HCL, 0.1 M EDTA) and stored it at –20°C.

### DNA Amplification

We amplified DNA using methods previously described (Carrell and Frank, 2014). Briefly, we used a nested PCR using a thermocycle profile with reduced PCR cycles to minimize PCR bias (Jiao et al., 2006). For the initial PCR, we used the chloroplast excluding primer 16S 799f (AACMGGATTAGATACCCKG) and 16S 1492r (TACGGHTACCTTGTTACGACTT) which resulted in a mitochondrial product of about 1000 bp and a bacterial product of about 750 bp as described by Chelius and Triplett (2001). We then separated the bacterial product from the mitochondrial product and extracted the bacterial product using E-Gel^®^ SizeSelect^TM^ Gels (Life Technologies, Carlsbad, CA, USA). We then used the extracted bacterial product as a template for PCR using the thermocycle profile described by Jiao et al. (2006) and the Golay-barcoded primer set 799f and 1115r (AGGGTTGCGCTCGTTG) (Redford et al., 2010). We performed a negative PCR control the same way, but with no template DNA added (but this control was not sequenced). We cleaned the final product with the QIAquick PCR cleanup kit, quantified the DNA concentration using Nanodrop, and pooled equal amounts of all 27 samples for pyrosequencing. The pooled product was sequenced at the Environmental Genomics Core Facility at the University of South Carolina for pyrosequencing on a 454 Life Sciences Genome Sequencer FLX machine.

### OTU Generation

We analyzed and processed the sequences using the QIIME package (Caporaso et al., 2010b). We quality filtered the sequences (minimum quality score of 25, minimum length of 200 bp, and no ambiguity in primer sequence) and assigned them to their corresponding sample by the barcode sequences. We removed sample EU (CR tree E, upper canopy) due to an insufficient number of sequences (59 sequences). One GS sample (tree A, middle canopy), was dominated by *Staphylococcus epidermis*, a common member of the skin microbiota, at high relative abundance (40%), and we discarded it due to likely contamination. We clustered the remaining sequences into OTUs using UCLUST, with a minimum coverage of 99% and a minimum similarity of 97%. A representative sequence was chosen for each OTU by selecting the longest sequence that had the highest number of hits to other sequences of that particular OTU. We detected chimeric sequences with ChimeraSlayer and removed them before taxonomic analysis (Edgar et al., 2011). We aligned representative sequences using PyNAST (Caporaso et al., 2010a) against the Greengenes core set (DeSantis et al., 2006). We made taxonomic assignments for the representative sequences using the Ribosomal Database Project (RDP) classifier (Wang et al., 2007) with greengenes representative set of sequences as reference. We removed sequences classified as “Chloroplast” (0.5%), “Mitochondria” (10%), or “Unassigned” from the alignment. We generated heatmaps using in-house perl/perl Tk scripts. We identified core OTUs using the script compute_core_microbiome.py in QIIME.

### Community Analysis

To evaluate communities at an equal sequencing depth, we rarified all samples to the lowest number of sequences occurring in a sample (594). We inferred an approximate maximum-likelihood phylogeny with FastTree (Price et al., 2009). We constructed unweighted and weighted UniFrac distance matrices from the phylogenetic tree to analyze dissimilarity of sample communities (Lozupone and Knight, 2005). To analyze the strength and statistical significance of sample groupings, we used Anosim and PERMANOVA as implemented in QIIME. We used the Kruskal–Wallis test as implemented in QIIME to determine whether differences in the relative abundances of individual bacterial taxa across sample types were significant.

### Phylogenetic Tree

To build a phylogenetic tree of the Alphaproteobacteria in our dataset, we created a dataset that contained only OTUs corresponding to Alphaproteobacteria present more than 50 times in our samples (34 OTUs total). First, we used this dataset as a query for BLAST searches against the NCBI 16S rRNA and GenBank non-redundant (nr) databases to identify the five top matching isolates or uncultured taxa that matched each OTU at or above 96% identity. We added matching sequences to our dataset, and aligned the sequences using infernal (Nawrocki et al., 2009). We removed highly variable regions and gap-only sites from the alignment using the filer_alignment.py script in the QIIME package and trimmed it to the ∼300 nucleotides covered by our 16S rRNA pyrosequences. We used RAxML (Stamatakis et al., 2005) to infer a maximum likelihood tree with 1000 bootstrap replicates, and plotted it using the Interactive Tree of Life tool (Letunic and Bork, 2011).

## Results

A total of 26 out of our 27 samples were successfully amplified and sequenced, and the negative PCR control was blank. One CR sample (from the Central CA location) had only 59 sequences, and was discarded from further analysis. We also removed one sample due to likely contamination (see Materials and Methods), giving us eight sequenced samples from GS, eight sequenced samples from the Central CA CR population, and nine sequenced samples from the Northern CA CR population (25 sequenced samples total). The samples yielded an average of 1741 sequences after plant DNA was removed. Rarefaction plots did not saturate, indicating that we under-sampled the bacterial communities at the 97% OTU level (data not shown). The sequence data have been submitted to the GenBank databases under project accession number SRP045230.

**FIGURE 1:**
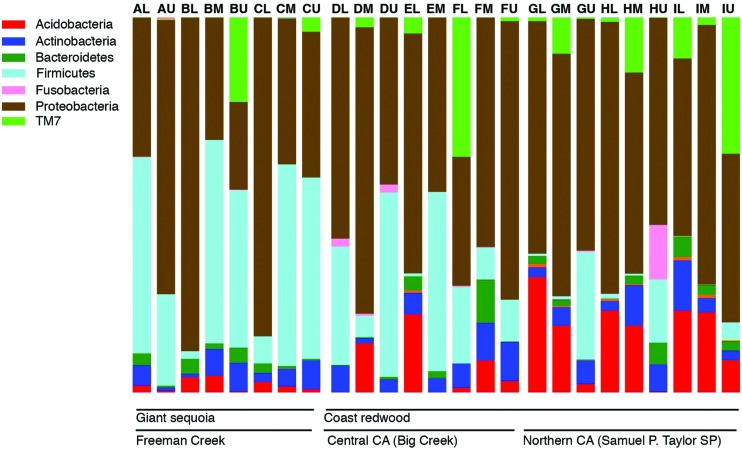
**Bar chart showing the relative abundance of major phyla in all the samples as percentages of all 16S rRNA gene sequences recovered in our foliage samples.** Each bar represents a sample, and letters A–I indicate individual trees (nine total), while L, M, and U indicate the canopy location from which the sample was taken (lower, middle, or upper).

Across all samples, the most abundant phyla in all samples were Proteobacteria and Firmicutes, followed by Acidobacteria, Actinobacteria, TM7, and Bacteroidetes. The relative abundance of bacterial phyla varied among samples, but Proteobacteria or Firmicutes dominated most samples (**Figure 1**). Firmicutes were significantly more abundant in GS (35% of the sequences on average) than in CR (13% of the sequences on average; *P* < 0.05), and significantly different across locations (35, 22, and 6% of sequences from Freeman Creek, Central CA, and Northern CA, respectively; *P* < 0.005). Among the Proteobacteria, Alphaproteobacteria was the most prominent class, followed by Betaproteobacteria. Among the Firmicutes, Bacilli dominated.

**FIGURE 2:**
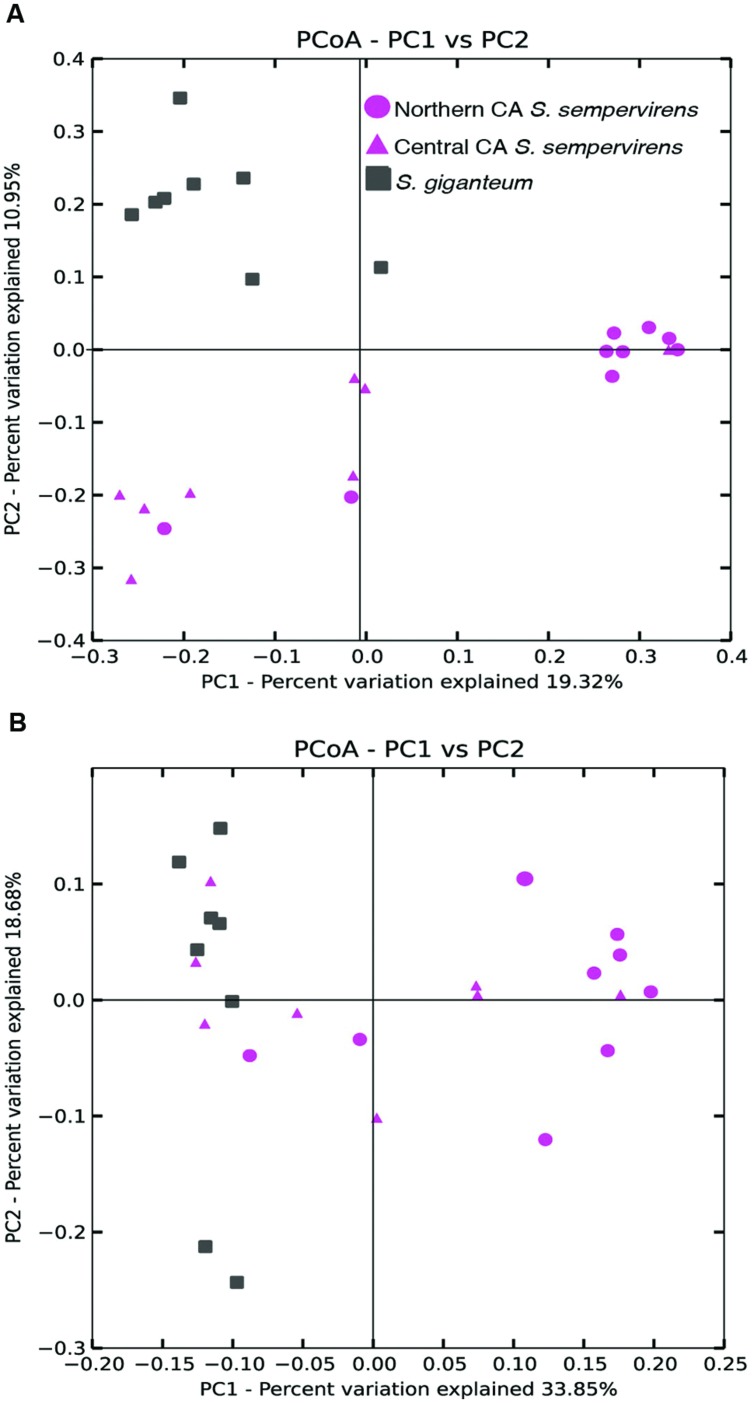
**Principal coordinate analysis (PCoA) of the (A) unweighted and (B) weighted UniFrac distance matrices.** Points that are closer together on the ordination have communities that are more similar. Each point corresponds to a sample, and shapes correspond to host tree populations. Coast redwood (CR) samples are shown in pink, and giant sequoia (GS) samples are shown in gray.

We used principal coordinate analysis (PCoA) of weighted and unweighted UniFrac distances to investigate patterns of separation between endophyte communities in samples from the different locations (**Figure 2**). We found that unweighted UniFrac identified clustering by species (**Figure 2B**: Permanova: Pseudo-F statistic = 3.2009, *P* = 0.001; Anosim: *R* = 0.4557, *P* < 0.001). The CR communities formed two clusters that largely separated Northern and Central CA populations (Permanova: Pseudo-F statistic = 2.7375, *P* < 0.001; Anosim: *R* = 0.5349, *P* < 0.001), with some overlap. When we took into account the relative abundance of taxa in addition to the presence of bacterial taxa (using weighted UniFrac distance matrices) clustering by species still occurred (**Figure 2B**: Permanova: Pseudo-F statistic = 6.12, *P* = 0.001; Anosim: *R* = 0.5464, *P* < 0.001).

**FIGURE 3:**
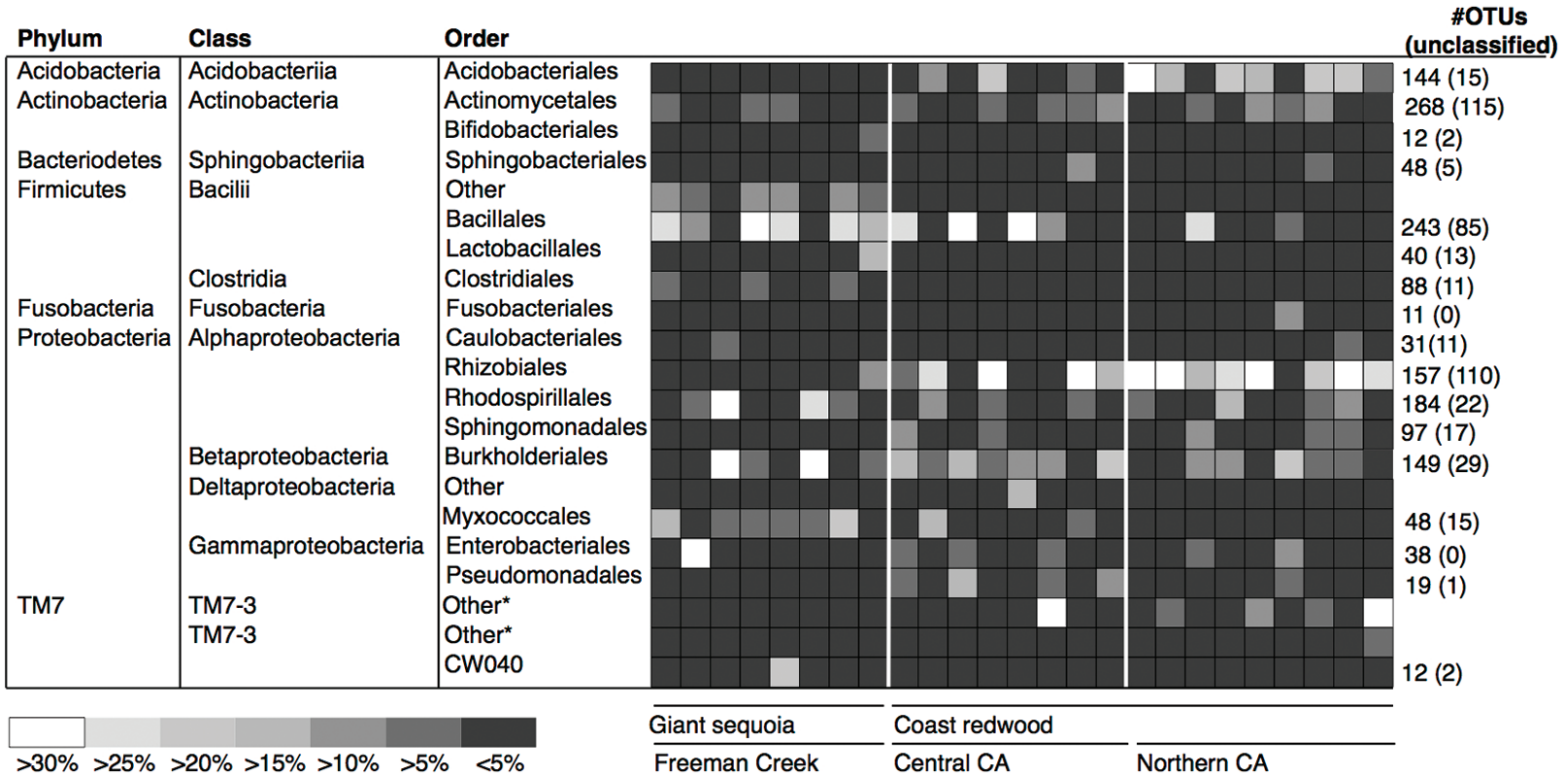
**Heatmap showing the 20 most dominant orders in our dataset and their average relative abundances as percentages of all 16S rRNA gene sequences recovered in our foliage samples, along with the total number of operational taxonomic units (OTUs) in each order.** The number of OTUs in each order that could not be classified below the order level is shown within parenthesis. Color tones range from white to dark gray to indicate the highest to lowest relative abundance values.

Next, we examined our sequences for high-level taxonomic groups that consistently dominated our samples within or across locations. There were no significant differences in the distribution of the most common bacterial orders between locations or tree species (**Figure 3**). Acidobacteriales, Actinomycetales, Bacillales, Rhizobiales, Rhodospirillales, and Burkholderiales were the most abundant and diverse orders, each represented by over 100 OTUs, many of which could not be classified below the order level (e.g., 43% of the Actinomycetales OTUs, 35% of the Bacillales OTUs, 70% of the Rhizobiales OTUs).

**FIGURE 4:**
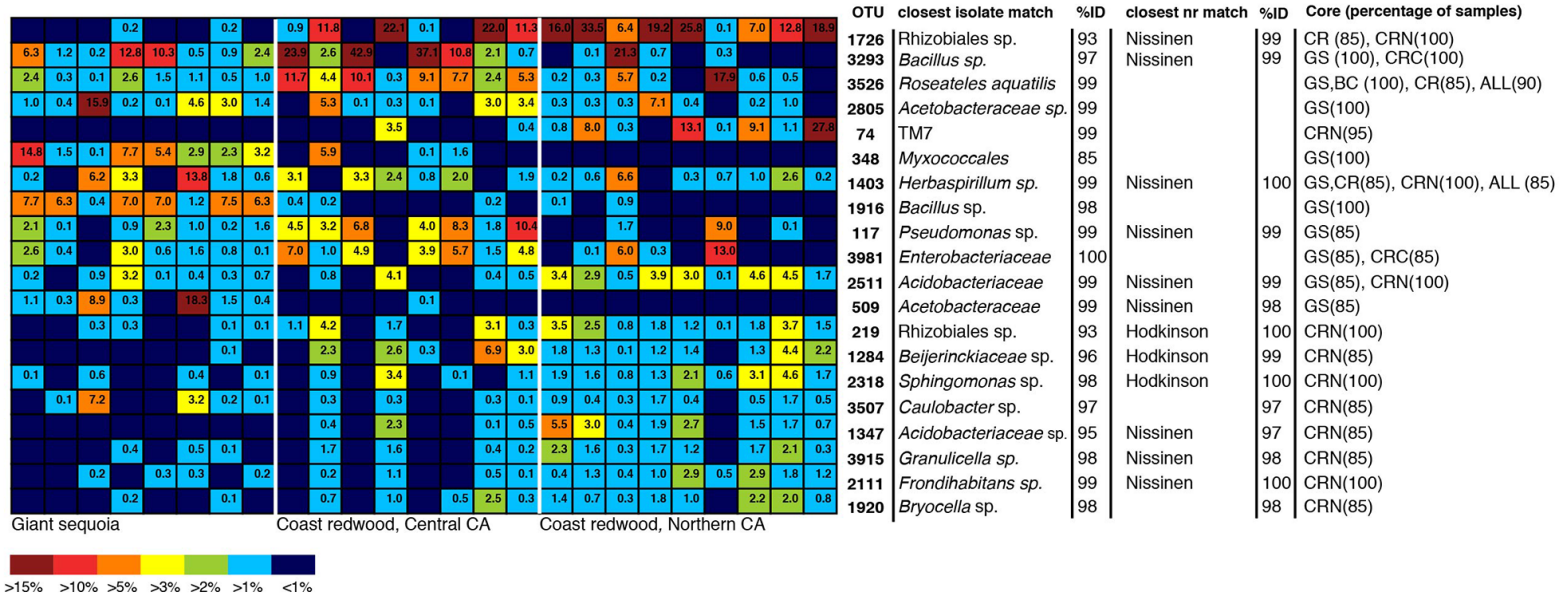
**Heatmap showing the 20 most dominant OTUs in our dataset, along with best matches in the GenBank 16S rRNA database, an indication if the top GenBank nr match was a sequence from the Hodkinson et al. (2012) or Nissinen et al. (2012) studies, and their status as core OTUs across all samples (ALL), GS samples, CR samples, Coast redwood from Northern CA (CRN) or Coast redwood from Central CA (CRC).** Within parenthesis, the percentage of samples above which the OTU is present. Color tones range from warm (red) to cool (blue) to indicate the highest to lowest relative abundance values.

**FIGURE 5:**
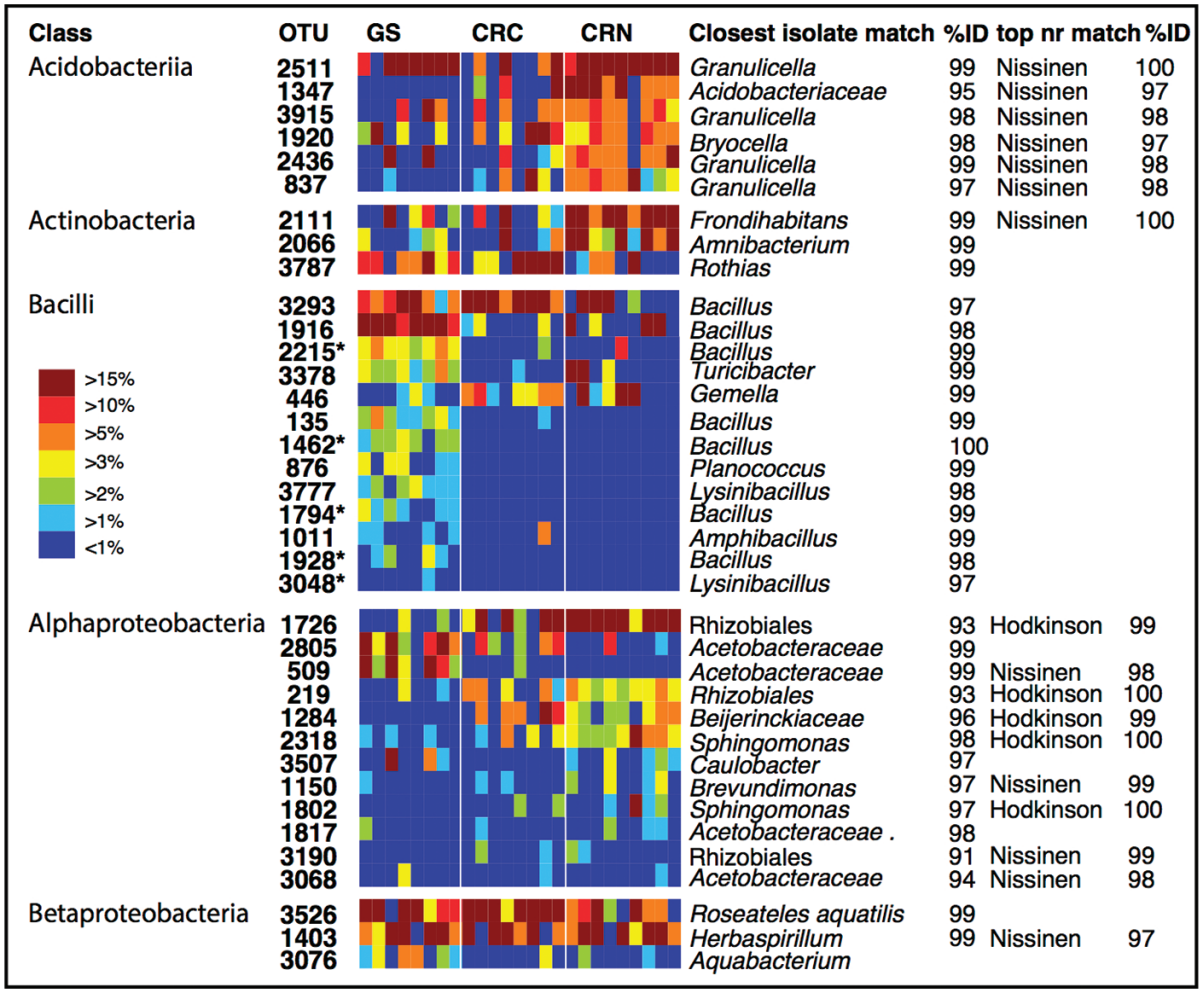
**Individual OTU heatmaps for dominant and diverse classes, along with best matches in the GenBank 16S rRNA database and an indication when the top match to GenBank nr was to sequences from the Hodkinson et al. (2012) or Nissinen et al. (2012) studies.** Here, colors represent the relative abundance of each OTU as a percentage of the total OTUs within each class. Color tones range from warm (red) to cool (blue) to indicate the highest to lowest relative abundance values. GS: Giant sequoia; CRN: Coast redwood from Northern CA; CRC: Coast redwood from Central CA. Five OTUs which were significantly more common in giant sequoia than in CR are marked with an asterisk.

To identify dominant members of the endophyte communities and their distribution across samples, we looked for OTUs that were present in both high relative abundance and were consistently present (i.e., in >85% of samples) within a species or population (hereafter referred to as core OTUs). **Figure 4** shows the overall 20 most common OTUs in our dataset, along with their status as core OTUs across all samples or within a population. In addition, to capture the dominance and variation of OTUs within major groups (**Figure 4**), but that were not necessarily among the 20 top OTUs in the entire dataset, we did this separately for the OTU-rich classes Acidobacteria, Actinobacteriia, Bacilli, Alphaproteobacteria, and Betaproteobacteria (**Figure 4**). The results are shown in **Figure 5**, where the relative abundance of each OTU is calculated as the percentages of total OTUs within each class. In both cases (**Figures 4** and **5**), the resulting OTU sequences were used to query the NCBI 16S rRNA and nr databases for matches to isolates or uncultured sequences described in peer-reviewed manuscripts.

In contrast to the endophyte communities from high elevation pines in our previous study, where single AAB OTUs made up at least 15% in Engelmann spruce and 19% in limber pine (Carrell and Frank, 2014), no single taxon was consistently present with a relative abundance above 15% across the samples from within a site. The most common OTUs belonged to genera previously identified as endophytes, e.g., *Bacillus*, *Herbaspirillum*, *Pseudomonas*, and *Acetobacteraceae* (Elbeltagy et al., 2001; Cocking et al., 2006; Bacon and Hinton, 2011; Bordiec et al., 2011; **Figures 4** and **5**). Many of our dominant OTUs in the classes Acidobacteriia and Alphaproteobacteria (but not *Bacillus*) were most similar to sequences from one of two particular studies; a study of lichen-associated bacteria (Hodkinson et al., 2012), and a study of endophytes of the cold-tolerant arctic plants Alpine sorrel (*Oxyria digyna*), pincushion plant (*Diapensia lapponica*), and highland rush (*Juncus trifidus*) (Nissinen et al., 2012; **Figures 4** and **5**).

The most common OTU in our dataset (1726, **Figure 4**) was present in 85% of all CR samples, and in 100% of samples from the Northern CA population, where it was found in high relative abundance (6–34%) in all but one sample. This OTU is not closely related to any known isolate, but shares 99% identity to uncultured clones in the Lichen-Associated Rhizobiales-1 (LAR1) lineage. Taxa in this lineage are prevalent and recurring in the lichen microbiome (Hodkinson and Lutzoni, 2009; Hodkinson et al., 2012). While this OTU was not completely absent from our GS samples, it was only present in a few samples, and only at low relative abundance (**Figure 4**). In GS, several top OTUs were present across all samples (i.e., OTU 3293, 3526, 2805, 348; **Figure 5**). OTU 3293, which belongs to the genus *Bacillus*, was also present in all CR samples from the Central CA population; in a few of the samples at high relative abundance (20-40%; **Figure 4**). AAB, the family that was found to recur at high relative abundance in the subalpine conifers (Carrell and Frank, 2014), did not consistently dominate the foliar endophyte community of CR and GS, although taxa belonging to this group were present in many samples. For example, OTUs 2805 and 509 (**Figure 4**) belong to this group. OTU 2805 was found in the majority of samples from both species, while OTU 509 was absent from most of the CR samples (**Figure 4**). Also notable, half of the sequences from one of the GS samples fell within an OTU with 99% identity to database sequences from to the insect symbiont *Sodalis glossinidus*.

We looked for dominant OTUs that were significantly more common in a particular location and/or species, but found no significant differences in the distribution of the 20 most dominant OTUs between locations or tree species (Kruskal–Wallis). Only five *Bacillus* OTUs that were not among the most dominant overall, but which were dominant within the class *Bacilli*, were significantly more common in GS than in CR (indicated in **Figure 5**).

**FIGURE 6:**
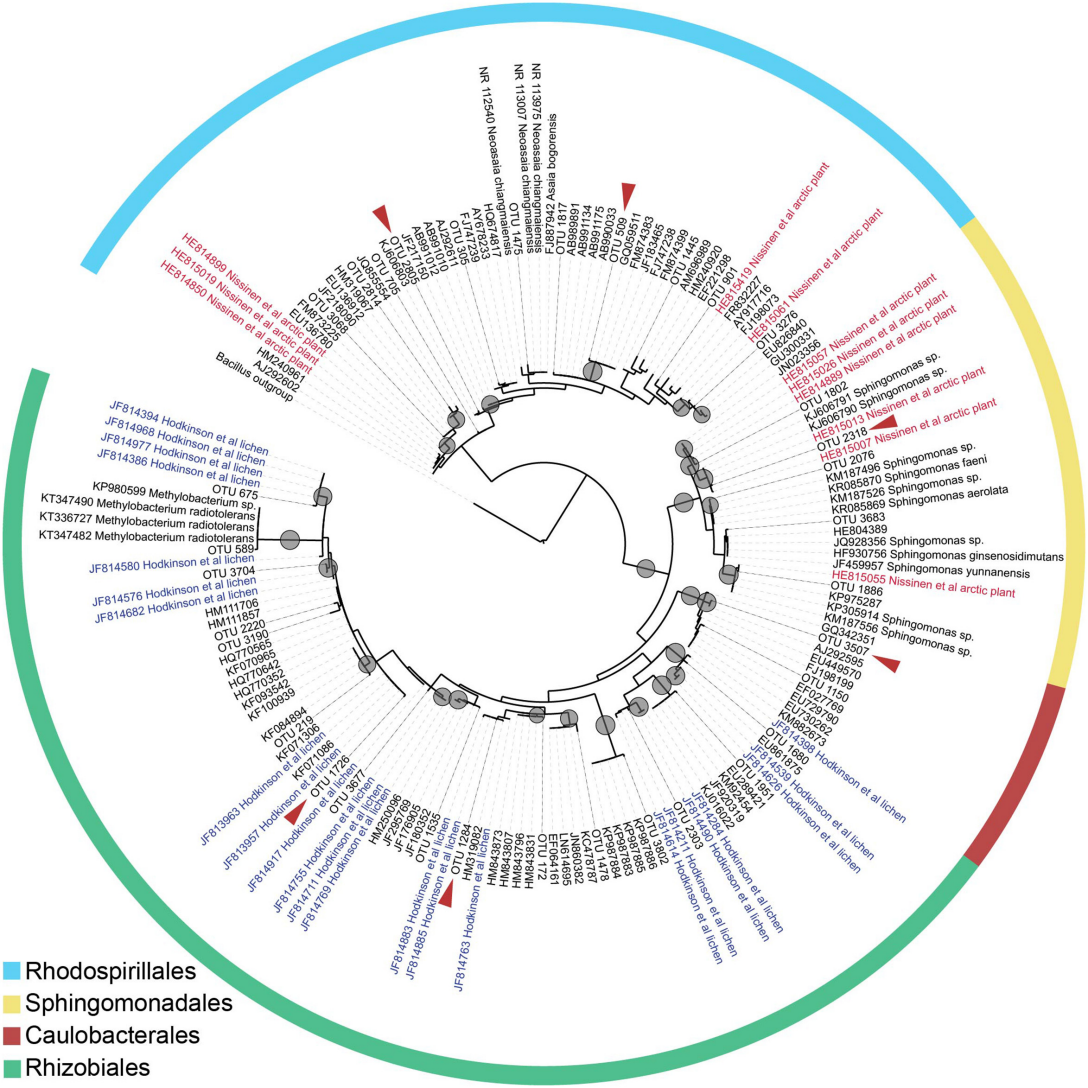
**Maximum likelihood tree inferred using the Alphaproteobacterial sequences in our dataset that occur above 50 times total.** Nodes with bootstrap support at or above 80 are indicated with a gray circle. Taxa named ‘OTU’ and with terminal branches shown in solid lines are OTUs from our dataset. Other taxa are indicated by their GenBank accession number, and in the case of isolates of known species, by species name. Taxa from the Hodkinson et al. (2012) study of lichen-associated bacteria are marked ‘Hodkinson’ and appear in blue, and taxa from the Nissinen et al. (2012) study on endophytes of arctic plants are marked ‘Nissinen’ and appear in red. A red arrow indicates that the OTU is among the 20 most abundant in the dataset (**Figure 4**).

To gain better taxonomic resolution for dominant Alphaproteobacterial OTUs (such as those belonging to LAR1 and AAB discussed above), we constructed a maximum likelihood phylogenetic tree from the Alphaproteobacterial sequences occurring more than 50 times in our samples, along with similar sequences in GenBank (≥96% identity). The phylogeny is shown in **Figure 6**. All Rhodospirillales sequences fell within the family *Acetobacteraceae* but could not be classified below the family level. Many were similar to sequences from Nissinen et al.’s (2012) study on arctic plants. Similarly, Rhizobiales sequences fell in uncultured lineages with the majority putatively in the LAR1 lineage commonly associated with lichens (Hodkinson et al., 2012). This includes some of the most common OTUs in our dataset (e.g., 1726 and 1284), which fell within clades together with LAR1 sequences. The sequences classified as belonging to the order Sphingomonadales also had matches to sequences from the study on arctic plants (Nissinen et al., 2012). While several of the Sphingomonadales OTUs were closely related (≥97% identity) to isolated bacteria (in the genus *Sphingomonas*), only one OTU in the Rhizobiales was closely related to known isolates (in the genus *Methylobacterium*). Similarly, only one OTU in the Rhodospirillales was closely related to an organism that has been cultured (in the genus *Neoasaia*).

## Discussion

The four phyla that dominated the CR and GS communities in this study— Proteobacteria, Firmicutes, Acidobacteria, Actinobacteria —are the same that constitute the majority of bacterial communities associated with the high elevation pines limber pine and Engelmann spruce (Carrell and Frank, 2014). This suggests that lineages within these phyla may be adapted to the conifer foliage endosphere and to the plant interior in general. These phyla have been found to dominate the endophyte communities of various plants (Gottel et al., 2011; Lundberg et al., 2012; Schlaeppi et al., 2014; Shen and Fulthorpe, 2015). Overall, the CR and GS communities were significantly different (**Figure 2**), but at the level of individual taxonomic lineages, few significant differences were observed. There were exceptions, such as the phylum Firmicutes, as well as individual OTUs within the Firmicutes, which were significantly more abundant in GS. It is possible that with more samples, we would see more significant differences in community composition between the two conifer species.

However, in contrast to the endophyte communities from limber pine and Engelmann spruce, no single taxon was consistently present above ∼15% within all samples from either CR or GS or from one of the three locations, and this result would likely not change with an increased sample size. In addition, AAB, while present in many samples, were not dominant taxa in CR and GS foliage. This difference is not due to batch effects associated with different sample processing or sequencing runs, since the samples in this study were prepared and sequenced at the same time as the Engelmann spruce (but not limber pine) samples from our previous study (Carrell and Frank, 2014). Thus, unless our DNA extraction method is not equally efficient in leaves from *Cupressaceae* and *Pinaceae* species, the differences observed here between trees in the two families are real, reflecting either the different environment in which the trees grow, the host species identity of the samples, or most likely, a combination of the two. The conifer leaf endophyte community could also be subject to seasonal or year-to-year variation (Shen and Fulthorpe, 2015), which might influence both the difference observed here between GS (which were sampled in August) and CR (which were sampled in October and November), and between the two conifer families, as the pines were sampled in September (Carrell and Frank, 2014). However, more recent data demonstrate that at least the relationship between AAB and pine is stable across years (Moyes et al., unpublished).

In both GS and CR, multiple OTUs were present in all samples from within a location (but at lower relative abundances than observed in the pines in our previous study). Such core OTUs may represent bacteria that are selected by the host, adapted to the environment inside the foliage, or present in high abundance in the source community (e.g., leaf surface, dust, or soil). Most notably, in CR foliage, an OTU belonging to the uncultured LAR1 lineage, which previously has only been described associated with the lichen symbiosis (Hodkinson et al., 2012), was present in all samples from the Northern CA population, and in most samples from the Central CA population. Our phylogenetic analysis of Alphaproteobacterial sequences, while limited by the length of the alignment (∼300 nt), suggests that our CR samples contain a wide a diversity of taxa belonging to LAR1 and/or other uncultured lineages in the Rhizobiales (**Figure 1**).

Interestingly, many of the dominant OTUs in the classes Acidobacteriia, Alphaproteobacteria, and Betaproteobacteria were most similar to uncultured endophytes of arctic plants (Nissinen et al., 2012), and several Alphaproteobacterial OTUs—in addition to those belonging to the LAR1 lineage—were most similar to uncultured bacteria associated with the lichen symbiosis (Hodkinson et al., 2012; **Figures 5** and **6**). Nissinen et al. (2012) demonstrated that many of their isolates from arctic plants were cold-tolerant. Endophytic mediation of plant tolerance to low-temperature stress has been reported in grapevine (Theocharis et al., 2012), however cold-tolerant endophytes do not necessarily provide cold-tolerance to the host plant.

Some possible functions of the CR and GS endophyte microbiome are protection against host biotic and abiotic stress, and N_2_ fixation. Several of the major and diverse bacterial groups in the CR and GS foliage (e.g., *Bacillus*, *Burkholderia*, Actinomycetes) are among those known to provide defense to plant hosts though, e.g., antimicrobial and antifungal activity (Mendes et al., 2011; Raaijmakers and Mazzola, 2012). Taxa belonging to the class Bacilli were present in all three populations, but were especially prominent in GS; several OTUs from this class were significantly more common in GS than in CR (**Figure 5**). Bacteria in the genus *Bacillus* show antagonistic activity to a wide range of potential phytopathogens, stimulate plant host defense, and are consequently exploited for biological control of plant diseases (Ongena and Jacques, 2008; Raaijmakers and Mazzola, 2012). For example, a *Bacillus pumilus* endophyte isolated from phloem of healthy lodgepole pine (*Pinus contorta*) is antagonistic against the fungal symbionts of the bark beetle (*Dendroctonus ponderosae*) (Adams et al., 2008). Likewise, Actinomycetes are well-known for their wide diversity of secondary metabolite production, many of which include antibiotic compounds (Tiwari and Gupta, 2012), including strains isolated from plants (Qin et al., 2011). Actinomycetes have been found to dominate the culturable antifungal population in the roots of Douglas fir (*Pseudotsuga menziesii*) (Axelrood et al., 1996). The *Burkholderiaceae* and *Pseudomonadacae* also harbor genera and species with activity against plant pathogenic fungi (Postma et al., 2010; Kwak et al., 2012; Suárez-Moreno et al., 2012).

The presence of these bacterial lineages in the foliage along with the lack of reported outbreaks of pests or diseases on CR and giant foliage is an incentive to further study their foliar bacterial microbiomes. For example, while redwood forests are one of the ecosystems most threatened by the oomycete sudden oak death agent *Phytophthora ramorum*, infection of CR is much lower than in co-occurring species such as tanoak (*Lithocarpus densiflorus)* and California–laurel (*Umbellularia californica*) and results in substantially less sporulation from infected needles (Davidson et al., 2008). Foliar endophytic fungi may contribute heterogeneity in defense chemicals that allows the giant trees to resist disease over centuries to millennia; unlike the host tree, their short life cycle should allow them to respond on ecological timescales to short-cycle pathogens and pests (Carroll, 1988). The bacterial community present within the foliage is another potential source of defense with high potential for spatial and temporal variability.

We previously hypothesized that AAB endophytes fix atmospheric N_2_ inside the needles of high elevation pines (Carrell and Frank, 2014). While AAB bacterial were only present at low relative abundance in CR and GS, we found that LAR1, a potential N_2_ fixing lineage associated with lichen thalli (Hodkinson and Lutzoni, 2009), was both consistently associated with CR (**Figure 5**), and represented by diverse taxa (**Figure 6**). Based on the phylogenetic affiliation of the *nifH* sequences from lichen, it has been hypothesized that lichen-associated bacteria in the LAR1 lineage fix and contribute atmospheric N_2_ to the lichen symbiosis (Grube et al., 2009; Hodkinson and Lutzoni, 2009). Endophytic N_2_-fixation may be a source of N_2_ to CRs, in addition to other suspected N sources such as fog (Ewing et al., 2009). Moreover, the presence of LAR1 taxa as endophytes in CR could reflect the high abundance of epiphytic lichens in the CR canopy (Williams and Sillett, 2007), which may share endophytic communities with their substrate tree. Redwoods, with their complex branch architecture and long lifespan, support large communities of epiphytic ferns, shrubs, and even trees (Sillett and Pelt, 2007; Williams and Sillett, 2007), all potential hosts of endophytic communities that could be shared with the redwood. Given the phylogenetic affinity of many of our dominant OTUs with endophytes from distant environments and hosts (i.e., arctic plants and lichens), the potential for endophyte sharing among partners in the redwood canopy ecosystem is probably high.

## Conclusion

The GS and CR trees we sampled did not host specific recurring bacterial taxa to the extent observed in high elevation conifers (Carrell and Frank, 2014); major OTUs were present but their relative abundance was more variable among samples. Bacterial groups known to be involved in plant defense were major members of the CRs and GS microbiomes, suggesting a potential role in host defense. Further studies using culturing protocols designed to maximize the recovery of specific bacteria such as Actinomycetes (Kaewkla and Franco, 2013) could be done to assess the antimicrobial and antifungal potential of bacteria isolated from surface-sterilized CR and GS foliage.

## Author Contributions

AC and AF conceived and designed the sampling and experiments. AC performed the DNA extraction, and PCR amplification. AC and AF analyzed the data and wrote the article.

## Conflict of Interest Statement

The Guest Associate Editor Mysore Tejesvi declares that despite having hosted a Frontiers Research Topic with the author Anna C. Frank, the review process was handled objectively. The authors declare that the research was conducted in the absence of any commercial or financial relationships that could be construed as a potential conflict of interest.
